# When the going gets tough: Extracellular vesicles transport lignin precursors and lignifying enzymes

**DOI:** 10.1093/plphys/kiae354

**Published:** 2024-06-27

**Authors:** Erin Cullen

**Affiliations:** Assistant Features Editor, Plant Physiology, American Society of Plant Biologists

Lignin is a stiff phenolic polymer and one of the most abundant chemicals produced by plants. It provides vascular plants with both structural support and a means to transport water, for example, allowing giant redwoods (*Sequoiadendron giganteum*) to grow beyond 100 m tall and draw water to their tips. Lignin monomers (monolignols) are produced in the cytosol and are polymerized in the cell wall. Laccase and peroxidase enzymes oxidize monolignols to generate phenolic radicals that randomly couple to form the final polymer. How lignin monomers are transported from the site of synthesis to the apoplast is an open question. Passive diffusion of monolignols across the plasma membrane due to a concentration gradient, generated by the oxidation of monolignols in the cell wall, is a recently proposed model (Perkins et al. 2022). Evidence also exists for active transport of monolignols; for example, the membrane-bound ATP-binding cassette (ABC) transporter *ABCG29* is reported to transport lignin monomers in *A. thaliana* (Alejandro et al. 2012).

**Figure 1.**
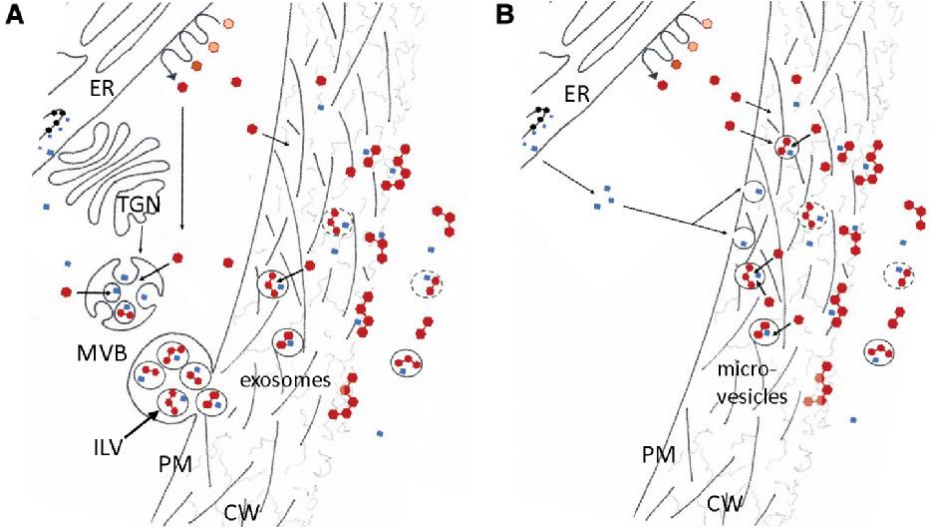
Two of the several hypotheses for the transport of proteins and monolignols to the apoplast are presented in panels A and B. Figure adapted from Kankaanpää et al. (2024). **A)** Multivesicular bodies (MVBs) encapsulate proteins and monolignols and fuse with the PM, releasing the lignin precursors into the apoplast. **B)** Lignin precursors move to the PM by diffusion or active transport, and microvesicles form at the PM.

Vesicular transport has been suggested as an additional mechanism of lignin transport to the apoplast. Extracellular vesicles (EVs) are vesicles surrounded by a phospholipid bilayer, which can transport proteins, nucleic acids, and lipids. EVs are present in every domain of life, and extensive research in mammalian systems has revealed involvement in intercellular signaling and human diseases such as cancer and autoimmune disorders. There are several possible ways that plant EVs can be assembled and secreted (Fig. 1). Suberin (a lipophilic polymer that is a component of some cell walls) deposition has recently been correlated with EVs (De Bellis et al. 2022), yet a role in lignin deposition has not been reported. Determining whether EVs are involved in lignin deposition is complex as EVs could also be generated by programmed cell death. Plant cell cultures are a method to bypass this issue, as EVs can easily be separated from the cell cultures.

In this issue of *Plant Physiology*, Kankaanpää et al. (2024) took advantage of previously established cell cultures of Norway spruce (*Piceca abies* L. Karst.) that release extracellular lignin (Kärkönen et al. 2002) to examine whether EVs are involved in the transport of lignin precursors and lignifying enzymes to the apoplast. The authors discovered that EVs were produced by cultures of *P. abies* in a range of morphologies and sizes, suggesting that different types of EVs may be present. Ultracentrifugation was used to separate the EVs from cultures and their contents examined through metabolomic and proteomic analyses. Lignin precursors, amino acids, and carbohydrates were identified in the EV samples. Moreover, the authors found evidence that radical coupling of monolignols occurred in these vesicles. Interestingly, salicylic acid was also identified in EV samples, which has been shown to induce EV production in *Arabidopsis thaliana* (Rutter and Innes 2017).

Proteomic analysis revealed the lignin polymerizing enzymes, laccases and peroxidases, were present in EV samples. Several of these enzymes are known to coexpress with enzymes involved in monolignol biosynthesis both in plant cell cultures that generate extracellular lignin and in vivo in trees. Three dirigent proteins were also identified in the EV samples, which may act as guides for lignin polymerizing enzymes. Interestingly, ABC and major facilitator superfamily transporters were also identified, and the authors hypothesize that these may act in vesicle loading. Given that the EV cargo contained cell wall–associated proteins, these findings complement a growing number of studies that suggest plant EVs are involved in cell wall remodeling (Regente et al. 2017).

The authors further interrogated the proteomics data to assess the types of plant EVs present in *P. abies* cultures. Previous studies have indicated several mechanisms may underlie the formation and secretion of EVs. For example, multivesicular bodies are generated from the *trans*-Golgi network, which can fuse with the plasma membrane (PM) and release vesicles (exosomes) into the apoplast (Fig. 1). Alternatively, microvesicles could form at the PM to encapsulate the lignin precursors, which have travelled to the PM by diffusion or active transport (Fig. 1). Tetraspanin proteins are located on the outer membrane of exosomes and microvesicles. The authors detected a putative tetraspanin in the proteomics data, which suggests that either or both types of EVs could exist in *P. abies* cultures.

Overall, Kankaanpää and colleagues show for the first time, to our knowledge, that EVs generated by *P. abies* cultures transport both lignin precursors and the enzymes needed for their polymerization, which can take place inside EVs. This raises many stimulating questions for the burgeoning field of plant EV research: for example, what is the lipid composition of the phospholipid bilayer of the EV, and how do its properties effect the diffusion of phenolic compounds? What is the environment inside EVs (e.g. pH), and how does it affect lignin polymerization? How is the cargo of the EV unloaded into the apoplast? Furthermore, this work will inform future studies of the role of EVs in lignin deposition in a native context, for example, during xylem development in trees.
